# Integrating Structured Digital Tools with the Traditional Hands-on Puzzle Method for Teaching Tooth Morphology: A Comparative Study of Educational Outcomes

**DOI:** 10.3390/dj12080270

**Published:** 2024-08-22

**Authors:** Caroline Elisabet Markholm, Helene Lindén Overskott, Amer Sehic, Tor Paaske Utheim, Qalbi Khan

**Affiliations:** 1 Institute of Oral Biology, Faculty of Dentistry, University of Oslo, 0316 Oslo, Norway; carolem@student.odont.uio.no (C.E.M.); heleneld@student.odont.uio.no (H.L.O.); qalbi.khan@odont.uio.no (Q.K.); 2 Department of Plastic and Reconstructive Surgery, Oslo University Hospital, 0424 Oslo, Norway

**Keywords:** dental anatomy, digital learning, digital videos, tooth anatomy, tooth identification

## Abstract

The study of tooth morphology is a critical component of the dental curriculum, highlighting the importance for dental students to acquire comprehensive and detailed knowledge of the complex structure of teeth. This study compared the educational outcomes of two student cohorts in a tooth morphology course, using traditional methods for the control group and additional digital video-based resources for the experimental group. We hypothesized that early integration of digital resources would significantly reduce the learning time. We retrospectively analyzed two groups of Master of Dentistry students. The control group (42 students) was taught using the traditional ‘tooth puzzle’ method, while the experimental group (42 students) supplemented traditional teaching with digital video-based tools developed by our department. Both groups’ curricula culminated in a practical post-course test requiring the identification of 40 teeth, along with a mid-course test to track the students’ learning progression. The number and type of incorrectly identified teeth were recorded. The mid-course test showed significant performance differences. The control group had a median (Q1, Q3) value of faults of 12.0 (7.8, 20.5), whereas the respective value for the experimental group was 4.0 (0.0, 8.0) (*p* < 0.001). In the control group, none achieved faultless results, with only two students (4.8%) having at most two faults, and six students (14.3%) having no more than four faults. The control group averaged 13.5 faults per student, with 19 students (45.2%) failing the test. Conversely, the experimental group showed improved performance: 12 students (28.6%) had no faults, and 25 students (59.5%) had four or fewer faults. The experimental group averaged 5.2 faults per student, with only four students (9.5%) failing. By the end of the course, both groups achieved commendable results on the practical tooth identification test. The experimental group slightly outperformed the control group, though the difference was not significant. The median (Q1, Q3) values were 0.0 (0.0, 2.5) and 1.0 (0.0, 4.5) for the experimental and control groups, respectively (*p* = 0.372). The students using both traditional and structured digital video-based tools showed greater learning advancement than those using only the traditional ‘tooth puzzle’ method.

## 1. Introduction

A thorough knowledge of tooth anatomy is fundamental in various dental disciplines [1,2], requiring visualization and understanding of dental morphology [3]. Within the context of dental education, the significance of tooth morphology might not be immediately apparent to students, and the long-term retention of its complex details may diminish. Consequently, dental curricula have evolved, now offering a range of instructional approaches to accommodate diverse learning styles and enhance educational adaptability. Educational technology advancements have prompted a transition from traditional pedagogical methods to more engaging and interactive digital formats [4].

Didactic lectures continue to play a pivotal role in the teaching of tooth morphology, largely due to the utility of programs like PowerPoint that facilitate the creation of chronological and comprehensible presentations. These programs also introduce innovative ways to utilize videos, animations, and images [5,6]. Interactive e-learning tools enhance the educational experience by providing immersive and engaging environments for learners [7,8]. However, these methods are typically viewed as valuable supplements rather than replacements for traditional teaching methods [9,10,11]. To achieve a detailed understanding of anatomical structures, hands-on practical courses have been deemed essential. Such courses employ dental anatomy carvings and the use of plastic teeth models [4,12]. However, teaching the biological variations inherent in natural teeth poses a challenge when using plastic models or carvings. Consequently, courses that incorporate extracted teeth offer a significant educational advantage, provided that ethical and health concerns are adequately addressed [1,4,13]. The ‘tooth puzzle’ teaching method at the University of Oslo’s Institute of Oral Biology exemplifies a hands-on educational approach. This curriculum deeply engages students in the study of tooth morphology using real extracted teeth, thereby ensuring a comprehensive understanding of dental anatomy. This method combines both tactile and visual interaction to enhance learning outcomes [13].

Recent trends indicate a growing integration of e-learning elements in the teaching of tooth morphology, enhancing accessibility and versatility of educational content for a broader student audience across various teaching scenarios [4,14]. The modern flipped classroom model, which combines digital resources with practical coursework, has been recognized as beneficial for learning. In this model, students first encounter key concepts through brief lectures and subsequently engage in practical sessions, ideally in collaborative groups, to further explore and create the introduced topics [15,16,17]. We therefore argue that the most effective teaching methodology for tooth morphology is the one that successfully integrates elements of the flipped classroom with e-learning resources and a practical course employing the tooth identification puzzle method using extracted human teeth [18,19]. Accordingly, this study aims to compare the educational outcomes of two cohorts of students enrolled in a tooth morphology course over successive academic years, examining the impact of different teaching strategies. We hypothesize that implementing additional digital video-based resources early in the process enhances the students’ learning outcomes significantly.

## 2. Materials and Methods

**Figure 1:**
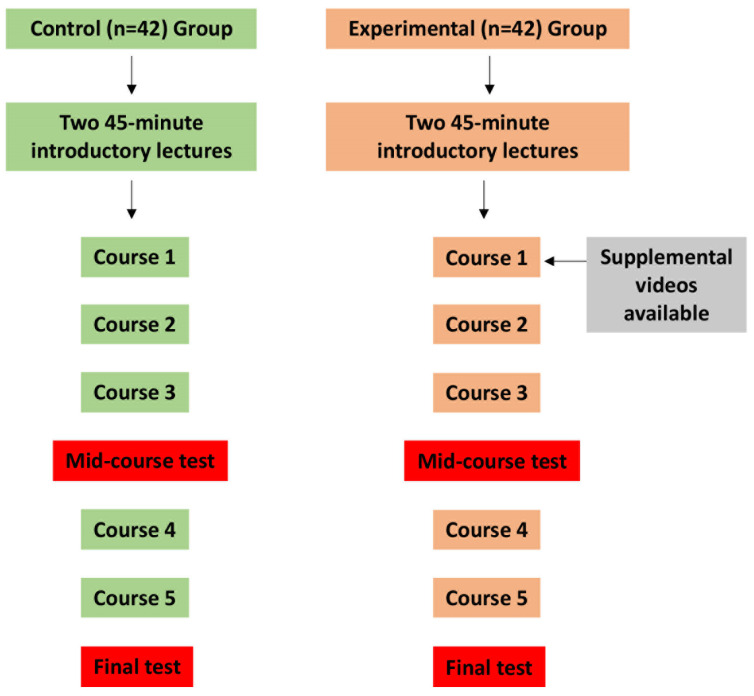
Study design and methodology. This flowchart depicts the study’s structure, contrasting two different pedagogical approaches. The control group, made up of 42 students, used the tactile ‘tooth puzzle’ method, which involved hands-on access to real teeth and comprehensive study materials. In contrast, an experimental group of the same size supplemented the conventional teaching with structured digital video-based tools developed by our department. Both groups underwent two tests: a mid-course test after the third course and a final test to assess their learning progression and outcomes.

**Figure 2:**
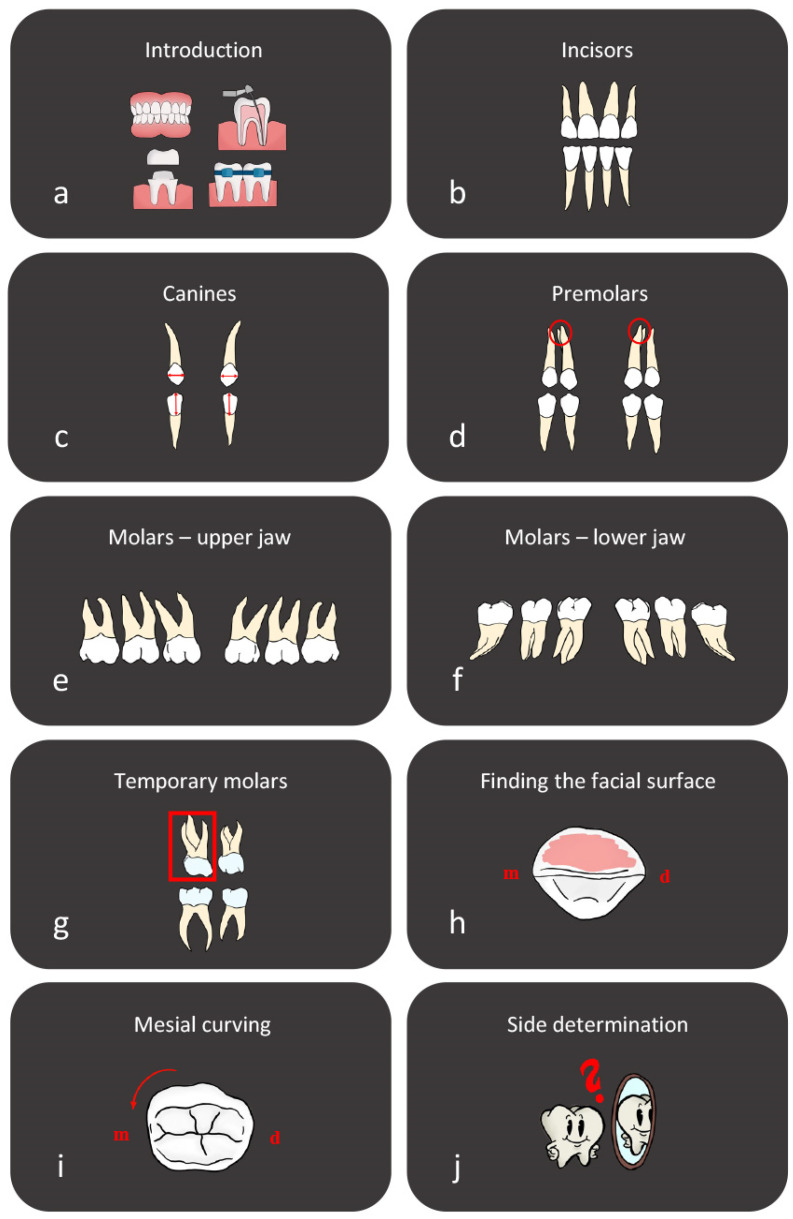
Digital video-based teaching tools. This figure highlights the digital video-based tools developed by our department to supplement the tooth morphology teaching method. A total of 10 videos, each lasting between 2 and 6 min, cover key topics in tooth morphology. The series begins with a general introduction video, followed by detailed presentations on the main tooth groups and their essential characteristics, including those of temporary molars. Additional videos focus on identifying the facial surface of the tooth and understanding the mesial curvature of the facial surface. The series concludes with an in-depth video on side determination, providing comprehensive guidance for students. These videos are designed to enhance traditional teaching methods, offering a robust multimedia resource to aid student learning and retention of complex dental concepts. m; mesial, d; distal.

This retrospective study aims to compare the educational outcomes of two cohorts of Master of Dentistry students in a dental anatomy course across sequential academic terms, differentiated by their instructional methods (Figure 1). In 2023, a control group of 42 students were taught using the ‘tooth puzzle’ teaching method [13], while in 2024, an experimental group of the same size supplemented the conventional teaching with structured digital video-based tools developed by our department (Figure 2). All participants were informed about the study and recruited from the Faculty of Dentistry at the University of Oslo, specifically from the second year of the Master of Dentistry program. The age and sociodemographic backgrounds of the students were relatively homogeneous, as most of the participants were between 20 and 25 years old, and sociodemographic differences in Norway are minimal. However, there was a notable gender disparity, with females comprising approximately 80% of the student population.

The ‘tooth puzzle’ method, as explicated in the literature [13], provided the control group with a regimented educational experience. It commenced with two 45 min lectures summarizing the course, followed by a 12 h hands-on segment spread across three weeks for tooth identification. The students interacted with sets of extracted teeth, composed of a complete series of 32 permanent teeth and 8 deciduous molars. These teeth, of undisclosed origin, were either provided to the Faculty of Dentistry by affiliated dental offices or collected from the student clinic and were approved for educational use. The students were instructed to adhere to general hygienic protocols, with no additional specific instructions. The teeth were preserved in 70% ethanol for several years in glass jars and thoroughly dried to eliminate any organic matter before use. The teeth sets, held in bags, were assigned to students based on material availability and preference; the students worked either alone or in small groups. The task involved using the FDI notation system to accurately position each tooth on a schematic diagram of the dentition. The control group had access to roughly 30 teeth sets and a comprehensive tooth morphology compendium but were not provided with additional digital resources by the educators, although they could utilize other e-learning supplements as needed. The 2024 experimental group began with two lectures and received the same compendium as the control group. Additionally, they were provided with structured digital video-based tools from the beginning of their learning experience (Figure 1 and Figure 2).

**Figure 3:**
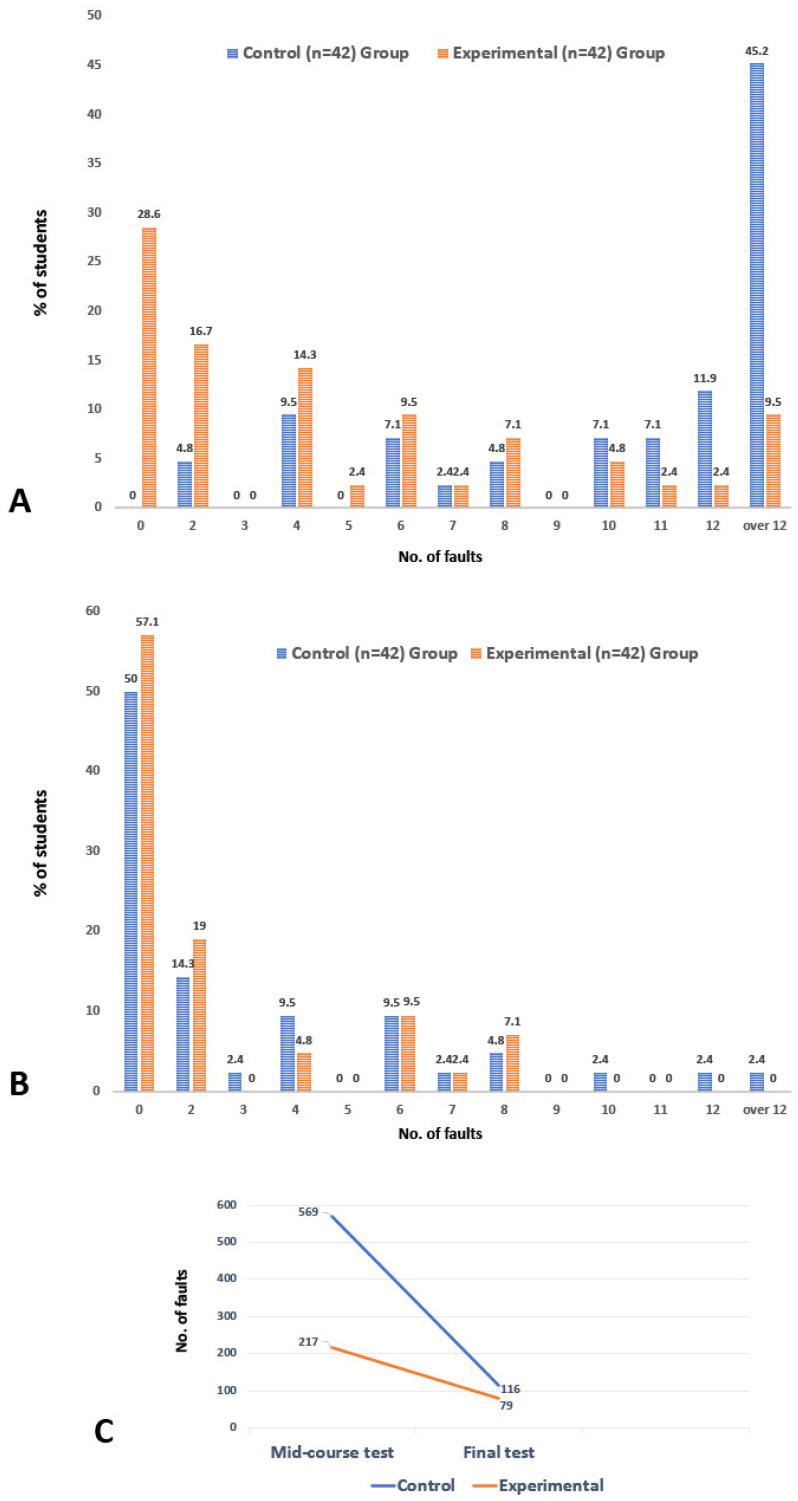
Comparative analysis of tooth identification exam results. This figure presents a thorough analysis of the mid-course (**A**) and final (**B**) test results from the tooth morphology course, comparing the performance of the control and experimental groups. It details the percentage of students in each group who made different numbers of errors in the identification tests. Furthermore, the total number of errors for both tests is displayed in (**C**). This visualization provides a clear view of the distribution of errors across the groups, offering valuable insights into the effectiveness of the respective teaching methods.

Both groups’ curricula culminated in a practical exam [13] requiring the identification of 40 teeth, supplemented by a mid-course test designed to track the students’ learning progression (Figure 1). During assessments, reference materials were not allowed, and a maximum of 12 misidentifications constituted the pass threshold. The number of incorrectly identified teeth was carefully documented. The collected data on the students’ performance were noted as the number of errors in both the control and experimental groups (Table 1). A comparison of the number of faults between the two groups was performed using the Mann–Whitney U test using IBM SPSS Statistics software (version 29). The percentage of students in each group, based on the number of errors made, was calculated using Microsoft Excel and displayed as a histogram in Figure 3. Additionally, the types of faults were recorded, and their percentages were calculated (Table 2).

## 3. Results

The collected data on student performance are detailed in Table 1 and Table 2 and visualized in Figure 3. These data indicate that the students in the experimental group, who used a combination of traditional and structured digital video-based learning tools, advanced more in their learning compared to those in the control group, as judged by the results from the mid-course test. However, at the end of the course, based on a practical tooth identification test, both groups achieved commendable outcomes, with the experimental group slightly outperforming the control group, although the difference was not substantial.

The mid-course test results revealed significant disparity in performance. The control group had a median (Q1, Q3) value of faults of 12.0 (7.8, 20.5), whereas the respective value for the experimental group was 4.0 (0.0, 8.0) (*p* < 0.001). Within the control group of 42 students, none achieved faultless results. A mere two students (4.8%) had at most two faults, and six students (14.3%) had no more than four faults. In total, the control group accumulated 569 faults, averaging 13.5 faults per student. Notably, 30 students (71.4%) had 10 or more faults, and 19 students (45.2%) failed the test by accruing more than 12 faults, with the highest individual fault count reaching 31 (Figure 3A, Table 1). In comparison, the experimental group showed a marked improvement in the mid-course test. Of its 42 students, 12 (28.6%) recorded no faults, and an impressive 25 students (59.5%) kept their faults to four or less. On the higher end, only 18 students (19.0%) had 10 or more faults, and merely 4 students (9.5%) failed the test due to more than 12 faults. In total, the experimental group recorded 217 faults, with an average of 5.2 faults per student, and the highest number of faults for an individual was 26 (Figure 3A, Table 1).

By the final test, however, both groups performed nearly equally well, with the experimental group again achieving slightly better results (Figure 3B, Table 1). The median (Q1, Q3) values were 0.0 (0.0, 2.5) and 1.0 (0.0, 4.5) for the experimental and control groups, respectively (*p* = 0.372). Of the experimental group, 24 students (57.1%) secured perfect precision with no faults, and no student had more than eight faults. The experimental group registered 79 faults in total, compared to the control group’s 116 faults. From the control group, only one student (2.4%) failed the test by committing more than 12 faults.

Upon the course’s conclusion, both the control and experimental groups demonstrated improved proficiency, though a detailed examination of the mid-course performance revealed notable differences in the frequency and types of tooth misplacement errors between the two groups. Specifically, 33.9% of teeth (569 out of 1680) were mispositioned by the control group, in contrast to a significantly reduced misplacement rate of 12.9% (217 out of 1680) observed in the experimental group. Further scrutiny of the data from the control group identified the central mandibular incisors as the most frequently misplaced teeth, accounting for 17.4% of all misplacement errors. This was followed by the second maxillary premolars at 15.6%, the first mandibular premolars at 12.0%, the second mandibular incisors at 9.3%, and the mandibular third molars at 9.0% (Table 2). Together, these five types of misplacements constituted 63.4% (361 out of 569) of the total misplacement errors for the experimental group, as detailed in Table 2. When evaluating the experimental group against the same criteria, these five teeth categories represented a smaller proportion of errors, amounting to 47.9% (104 out of 217) of the experimental group’s total misplacement faults. Notably, the experimental group’s error distribution pattern did mirror that of the control group, except for some minor differences in the first two categories (Table 2).

## 4. Discussion

Based on our experience, the tooth identification puzzle approach to learning tooth morphology provides substantial benefits for both instructors and students. This method is cost-effective, requiring around 14–16 h to complete. Students expressed high levels of satisfaction with the course in both formal and informal post-course evaluations. They particularly enjoy the gamified aspect, and importantly, they show significant improvement in their skills. While progress is initially slow due to the time needed to grasp the fundamental principles of tooth structure, descriptive nomenclature, and the distinctions among different teeth, the overall gains are notable [13]. However, recent findings indicate that adapting the well-established and effective ‘tooth puzzle’ method to a fully digital format, without hands-on interaction with real teeth, results in a significant decline in proficiency in tooth morphology [20]. On the other hand, the findings from this study indicated that students who utilized a combination of traditional and structured digital video-based learning tools showed greater advancement in their learning compared to those who used only the traditional ‘tooth puzzle’ method, as evidenced by the mid-course test results. By the end of the course, based on a practical tooth identification test, both groups achieved commendable outcomes, with the experimental group slightly outperforming the control group, though the difference was not significant.

The most effective way to teach the fundamental concepts of tooth morphology is through traditional lectures and practical sessions, supported by various advanced auxiliary teaching tools [19]. It is essential to integrate these innovative digital tools to enhance dental students’ learning experiences and motivation. These tools are most effective when combined with conventional teaching methods, such as lectures and courses. Although numerous supplemental teaching methods, including online tools and software programs, can help students to grasp certain curriculum components, they cannot replace the direct visual and tactile experience with extracted teeth [13]. The incorporation of e-learning, especially through interactive media, marks a significant shift from the traditional, linear lecture format, which is often characterized by low interactivity. A recent study regarding a revised curriculum that included e-learning components for dental morphology instruction concluded that, with proper structural support, students can learn more effectively through independent study than previously recognized in traditional lecture courses [3]. E-learning provides diverse methods for students to understand and review information. Numerous studies in higher education have shown that e-learning can achieve student performance outcomes equivalent to or better than those of classroom lectures, without compromising learning quality [21,22]. Additionally, recent findings indicate that e-learning strategies using computer-animated graphics for teaching human dental morphology are statistically as effective as traditional lecture methods [14]. Our results have demonstrated that in the beginning of our course, both the control and experimental groups progressed at a consistent rate as they developed a solid understanding of the fundamental principles of tooth structure and became familiar with dental terminology. Although both groups initially encountered challenges, the experimental group, which utilized structured digital videos to illustrate key principles and characteristics, demonstrated more significant improvement after this phase. During this time, the control group explored a range of digital resources on their own, utilizing materials like videos, images, and external e-learning platforms, which included digital atlases and specialized applications. However, this self-directed use of resources did not result in the same level of improvement observed in the experimental group. These results indicate that the structured digital videos and tools—tailored to suit our teaching method by our team and systematically presented to the students—have a more substantial impact compared to the ad hoc use of digital resources found independently on the internet.

The findings from this study align with previous research. Using software-assisted teaching in a dental morphology course has been proven to enhance student learning outcomes when it serves as a supplementary resource instead of being the main educational tool [23]. Earlier studies have shown that conducting waxing exercises at home, supplemented by detailed imagery and instructional videos from the 3D Tooth Atlas, led to positive results [24]. Although students comprehended the theoretical aspects of tooth morphology through webinars enhanced with the 3D Tooth Atlas, most favored traditional in-person classes for direct interaction with faculty and peers. This preference reflects the innate human desire for interpersonal connections. Our significant experience with the ‘tooth puzzle’ method further underscores the value of a collaborative learning setting. Such an environment offers a vital foundation for encouraging meaningful and dynamic exchanges between students and faculty, which is critical for a rich educational experience. This method supports both academic achievement and the holistic well-being of students.

Our analysis revealed several noteworthy points regarding the educational process. The control group, which followed a traditional, well-established teaching method, predominantly misplaced the central mandibular incisors, second maxillary premolars, first mandibular premolars, second mandibular incisors, and maxillary third molars. This outcome is consistent with prior evaluations of groups subjected to the same teaching method [13]. Conversely, the experimental group exhibited a similar pattern of faults, with a minor variation in the first two categories, showing more faults in the second maxillary premolars than in the central mandibular incisors (Table 2). These findings highlight the critical importance of the practical hands-on component of the course. Despite the experimental group having significantly fewer faults, the overall learning patterns were quite similar. This contrasts sharply with our previous study, which assessed the learning outcomes of a group that received solely digital instruction [20]. In that research, there was no consistent pattern observed in the group’s tooth misplacements; instead, the errors were randomly spread across various dental positions. This suggests that while digital tools can be beneficial, they should complement rather than replace hands-on practice to ensure comprehensive learning outcomes.

The students who did not attend all lectures and practical courses were excluded to ensure that all participants in both groups received the same teaching method. However, this study has some limitations, including the inclusion of only one experimental group and the participation of only two observers/teachers. Additionally, gender differences were not accounted for, as the higher number of female participants would have resulted in insufficient data on the male participants. Future research should place greater emphasis on investigating gender differences and considering the impact of external factors on the learning process.

## 5. Conclusions

An in-depth education in tooth anatomy is essential for dental students to address practical challenges. Engaging multiple senses and using interactive, hands-on learning experiences are crucial for a well-rounded education. Our study, despite its limitations, indicates that well-structured digital videos and tools tailored to complement hands-on teaching and systematically presented are more effective than the ad hoc use of independently found digital resources. This approach is particularly beneficial in the early learning stages, as shown by better performance in tooth morphology tests in the experimental group before the mid-course evaluation. However, given enough time, all students mastered the material, with no significant differences between groups by the course’s end. This suggests that structured digital resources can enhance early learning and may impact the time and resources needed to master the curriculum. Additionally, these tools reduce the reliance on anatomical specimens and support comprehensive tooth morphology education throughout dental training.

## Figures and Tables

**Table 1:** Comparative analysis of student performance by fault incidence. This table combines the performance data of students from the control and experimental groups, detailing the number and proportion of students, segmented by their fault incidences. Additionally, it aggregates the total number of faults per group, providing a measure of overall precision and accuracy in the course evaluations.

	Mid-Course Test				Final Test			
	Control Group		Experimental Group		Control Group		Experimental Group	
No. of Faults	No. of Students (%)	Total No. of Faults	No. of Students (%)	Total No. of Faults	No. of Students (%)	Total No. of Faults	No. of Students (%)	Total No. of Faults
0	0 (0%)	0	12 (28.6%)	0	21 (50%)	0	24 (57.1%)	0
1	0 (0%)	0	0 (0%)	0	0 (0%)	0	0 (0%)	0
2	2 (4.8%)	4	7 (16.7%)	114	6 (14.3%)	12	8 (19%)	16
3	0 (0%)	0	0 (0%)	0	1 (2.4%)	3	0 (0%)	0
4	4 (9.5%)	16	6 (14.3%)	24	4 (9.5%)	16	2 (4.8%)	8
5	0 (0%)	0	1 (2.4%)	5	0 (0%)	0	0 (0%)	0
6	3 (7.1%)	18	4 (9.5%)	24	4 (9.5%)	24	4 (9.5%)	24
7	1 (2.4%)	7	1 (2.4%)	7	1 (2.4%)	7	1 (2.4%)	7
8	2 (4.8%)	16	3 (7.1%)	24	2 (4.8%)	16	3 (7.1%)	24
9	0 (0%)	0	0 (0%)	0	0 (0%)	0	0 (0%)	0
10	3 (7.1%)	30	2 (4.8%)	20	1 (2.4%)	10	0 (0%)	0
11	3 (7.1%)	0	1 (2.4%)	11	0 (0%)	0	0 (0%)	0
12	5 (11.9%)	60	1 (2.4%)	0	1 (2.4%)	12	0 (0%)	0
over 12	19 (45.2%)	418	4 (9.5%)	88	1 (2.4%)	16	0 (0%)	0
	42 (100%)	569	42 (100%)	217	42 (100%)	116	42 (100%)	79

**Table 2:** Predominant top 5 categories of tooth misplacements at the mid-course test. This table provides a detailed enumeration of tooth misplacement instances, as identified in both the control and experimental groups during the mid-course evaluation. It presents the aggregate count of misplacements for each group and delineates the frequency and corresponding percentage of each identified category of misplacement.

	Control Group (*n* = 569)	Experimental Group (*n* = 217)
Type of Fault	No. of Faults (%)	No. of Faults (%)
Central mandibular incisors	99 (17.4%)	24 (11.1%)
Second maxillary premolars	89 (15.6%)	32 (14.7%)
First mandibular premolars	69 (12.0%)	21 (9.7%)
Second mandibular incisors	53 (9.3%)	15 (6.9%)
Mandibular third molars	51 (9.0%)	12 (5.5%)

## Data Availability

The original contributions presented in the study are included in the article, further inquiries can be directed to the corresponding author.
